# Molecular and phenotypic variations in *Eutetranychus orientalis* (Klein) populations from Saudi Arabia

**DOI:** 10.1371/journal.pone.0233389

**Published:** 2020-05-19

**Authors:** Jawwad Hassan Mirza, Muhammad Kamran, Amgad A. Saleh, Fahad Jaber Alatawi

**Affiliations:** 1 Acarology Research Laboratory, Department of Plant Protection, College of Food and Agriculture Sciences, King Saud University, Riyadh, Saudi Arabia; 2 Plant Pathology Laboratory, Department of Plant Protection, College of Food and Agriculture Sciences, King Saud University, Riyadh, Saudi Arabia; Imam Abdulrahman Bin Faisal University, SAUDI ARABIA

## Abstract

The oriental red spider mite, *Eutetranychus orientalis* (Klein) is a major pest of citrus in many countries including Saudi Arabia (SA). The morphological variations among the different populations of *E*. *orientalis* were reported. In the present study, phenotypic variations based on 40 different morphological characteristics were evaluated in 10 *E*. *orientalis* populations collected from different hosts and regions of SA. Further, ITS2-rDNA sequences were used to confirm the identity of these phenotypically varying populations. Phenotypic variations in all populations were found in the shape and length of dorsal setae, striation pattern between setae *d1* and *e1*, and leg chaetotaxy. The rDNA sequence analysis of these morphologically varying populations resulted in 10 different ITS2 Saudi haplotypes. The phenotypic and genetic variations were more related to the host plants rather than their geographic distribution. The *E*. *orientalis* population collected from *Phoenix dactylifera* was phenotypically distinct and genetically divergent. The populations collected from citrus species were also more phenotypically and genetically related to each other than to populations collected from non-citrus host plants. The haplotypes recovered from *Ziziphus* sp., *Morus* sp., and *Azadirecta indica* from different regions were grouped in the same sub-clade. Further, the ITS2 haplotypes of Saudi *E*. *orientalis* recovered from *Citrus reticulata* from Riyadh and Al Ula were 100% identical to the ITS2 haplotypes recovered from *Citrus* sp. from Israel. It is concluded that phenotypic variations exist among different populations of *E*. *orientalis* inhabiting different host plants. This species should be identified carefully by considering phenotypic intraspecific variations.

## Introduction

The oriental red spider mite, *Eutetranychus orientalis* (Klein), is a major pest of many economical shrubs and fruit trees including *Citrus* sp. and *Prunus* sp. They are widely distributed in many countries of Oriental, Afrotropical, and Palaearctic regions, including Saudi Arabia (SA) and have been found inhabiting more than 200 host plants [1–4]. Different morphological variations have been documented among and within different *E*. *orientalis* populations [5–9]; these morphological variations in *E*. *orientalis* resulted in misidentifications. Recently, the morphological characteristics of the *Eutetranychus* species were tabulated with descriptions and illustrations; in addition, seven *Eutetranychus* species as junior synonyms of *E*. *orientalis* were suggested based on phenotypic variations [4]. At the intraspecific genetic variation level, the DNA sequences of the internal transcribed spacer 2 (ITS2) were used to genotype nine *E*. *orientalis* populations from Israel [10]. It was reported that the morphological and genetic variations in *E*. *orientalis* populations could be caused by either vast geographic distribution or polyphagous feeding behavior of the species [11–14]. Molecular techniques have been used over the past two decades to study the intra / interspecific variations within and among populations / species. Further, DNA-based intra and interspecific variations have been used to identify spider mite species [10, 15] and resolve many other cryptic species [16, 17]. DNA barcoding using short DNA sequences such as ITS2 or mitochondrial Cytochrome Oxidase I (COI) regions is an effective tool to study intraspecific variations within and among different populations of a species [18–21].

The aims of the present study were to (1) assess the phenotypic variations in different populations of *E*. *orientalis* collected from different localities and host plants in SA and (2) confirm the intraspecific nature of these phenotypic variations through ITS2-rDNA.

## Materials and methods

### Mite collection

**Fig 1:**
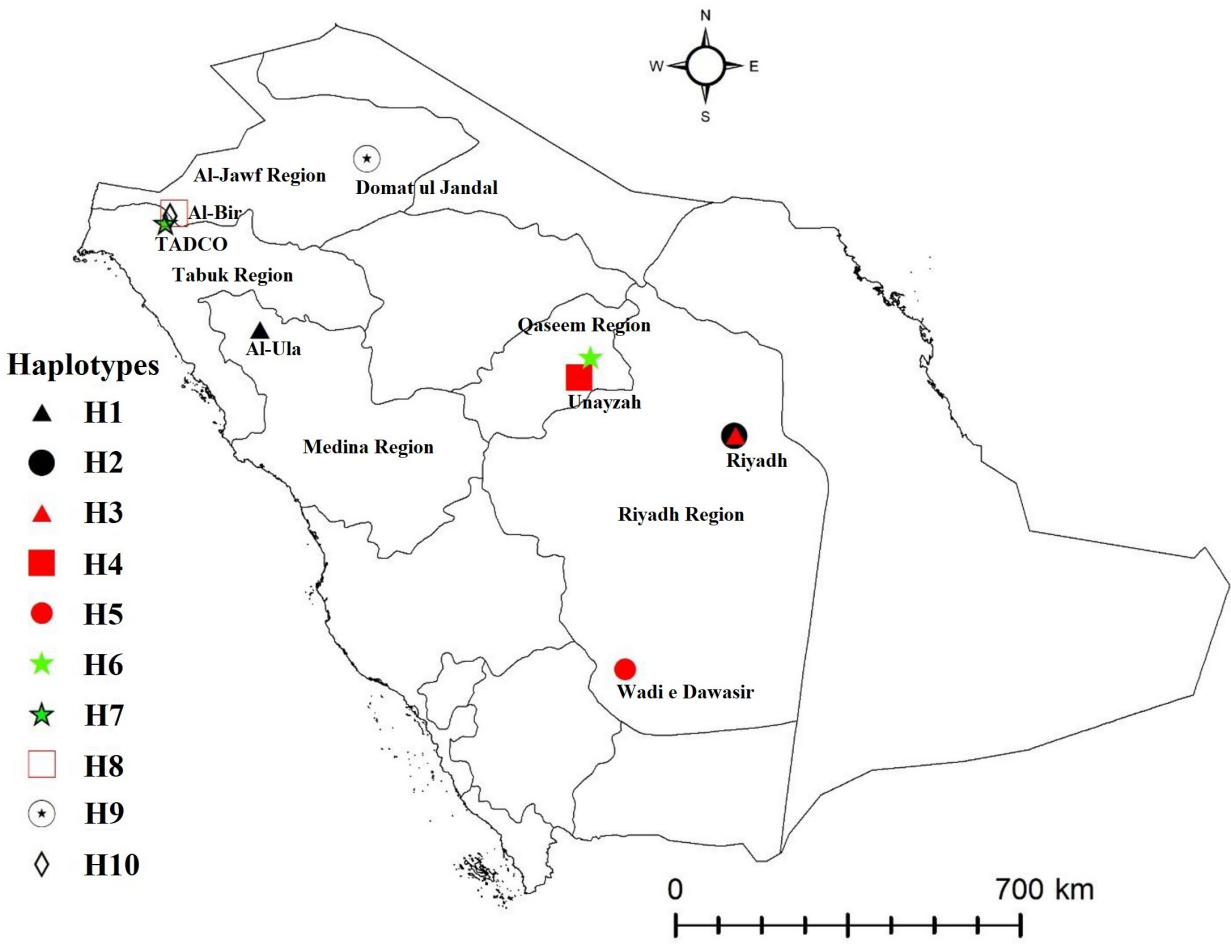
Geographic distribution of 10 different populations of *Eutetranychus orientalis* in Saudi Arabia (ArcGis 10.5., esri.com computer software).

This study did not involve any protected or endangered species. Populations of *E*. *orientalis* were collected from diverse host plants from different localities in six regions (Jizan, Makkah, Madina, Najran, Riyadh, and Tabuk) of SA during 2009–2018 (Table 1). The collection sites included agricultural farms and no specific permission was required for the collection of *E*. *orientalis*. A distribution map was generated using GPS coordinates of collection sites through ArcGis 10.5, Esri.com computer software (Fig 1). These mite specimens were collected by shaking vegetative plant parts over a white piece of paper. The mites visible under a hand-held magnifying lens and moving on the paper were picked using a camel hair brush and preserved in small vials containing 70% ethanol. These samples were brought to the Acarology laboratory, Department of Plant Protection, College of Food and Agriculture Sciences, King Saud University. Further, few adult females from each population were separated and preserved in 96% ethanol for molecular identification. Also, some adult mite females from each population were mounted on glass slides in Hoyer’s medium under a stereomicroscope (SZX10, Olympus, Tokyo, Japan). The species was identified under a phase contrast microscope (BX51, Olympus®, Japan) using the identification key [4].

**Table 1:** Geographical distribution and host plant information of Saudi *E*. *orientalis* populations.

Haplotype (H) Phenotype (P)	Locality/Region	GPS	Date	Botanical Name	ITS2 Fragment Size	Accession Number
1	Al-Ula/ Medinah	26°39.923'N, 37°55.032'E.	07 May 2017	*Ficus* sp. (in citrus orchard)	528	MK868097
2	King Saud University/ Riyadh	24°43.218'N, 46°36.478'E.	09 Dec. 2017	*Phoenix dactylifera*	530	MK868098
3	Agricultural Farm/ Riyadh	24°44.180'N, 46°37.317'E.	09 Dec. 2017	*Citrus* sp.	529	MK868099
4	Unayzah/ Qaseem	25°47.408' N, 43°45.647' E	03 May 2018	*Azadiracta indica*	534	MK868100
5	Wadi Dawasir/ Riyadh	20°27.476' N, 44°36.446' E	09 May 2018	*Ziziphus* sp.	511	MK868101
6	Unayzah/ Qaseem	26°9.397' N, 43°58.513' E	01 May 2018	*Morus* sp.	511	MK868102
7	TADCO/ Tabuk	28°37.380' N, 36°11.411' E	23 May 2018	*Citrus limon*	496	MK868103
8	Al-Bir/ Tabuk	28°47.887' N, 36°21.037' E	23 May 2018	*Citrus limon*	531	MK868104
9	Domat ul Jandal/ Al-Jawf	29°47.432' N, 39°52.680' E	26 May 2018	*Citrus* sp. (near *A*. *indica* trees)	528	MK868105
10	TADCO/ Tabuk	28°44.417' N, 36°16.748' E	22 May 2018	*Citrus* sp.	540	MK868106

### DNA extraction and amplification

Total genomic DNA was extracted from a single female, representing a population collected from 10 different locations (including seven different host plants), using a DNeasy mini kit (Qiagen Venlo, Netherlands). The data of the collection localities and hosts are provided in Table 1.Each extracted DNA sample was properly labelled with the name of the locality and host plant and the date of collection, and directly stored at -20°C. The concentration of total genomic DNA was evaluated using a Nanodrop spectrophotometer (Thermofisher Scientific, Waltham, Massachusetts, USA) and adjusted for PCR amplification. The PCR primers ITS2-forward (5'-GTCACATCTGTCTGAGAGTTGAGA-3') and ITS2-reverse (5'-GTARCCTCACCTRMTCTGAGATC-3') were used to amplify the ITS2 region of rDNA (Ben David et al. 2007).

The PCR reaction was performed in a 30 μL reaction volume with 15 μL GoTaq^®^ Green Master Mix (Promega, Madison, Wisconsin, USA), 0.75 μL of each 10 μM primer, 2 μL (approximately 20 ng) DNA template and 11.5 μL Nuclease free water (Promega). The PCR cycling conditions were as follows: denaturation step for 5 min at 94°C, followed by 35 cycles of 60 s at 94°C, annealing at 52°C for 90 s and 72°C for 60 s, and the final extension step at 72°C for 10 min. The PCR products were assayed in 1.2% agarose gel stained with Acridine Orange dye in 1× TAE buffer. These gels were later visualized under UV light using gel documentation system. Further, the PCR products were purified using a MoleQule-On PCR product purification kit (MoleQule On, Auckland, New Zealand).

### DNA sequencing and analysis

The purified PCR products were directly sequenced using the same primers used in the PCR amplification; the sequences were then run on ABI 3500 (Life Technologies, MD, USA). The obtained sequences were cleaned and analyzed using the BioEdit software [22]. Thereafter, the cleaned sequences were searched in the NCBI GenBank Database using BLAST. The ITS2 sequences of *E*. *orientalis* obtained in the present study and those retrieved from GenBank were aligned using a Clustal W multiple alignment tool. The final sequences were submitted to the NCBI database; the accession numbers of each population are provided in Table 1.

#### Phenotypic and phylogenetic analyses

The morphological variations between the collected populations were assessed based on different characteristics including length and shape of setae, length and chaetotaxy of legs, and dorsal striation patterns and shape of stylophore. Based on these phenotypic character, an UPGMA dendrogram was constructed using a Minitab 18 data analysis software (2010) (State College, PA: Minitab, Inc. (www.minitab.com). To assess genetic variations among 10 Saudi *E*. *orientalis* ITS2 sequences, neighbor-joining (NJ) trees were constructed using the MEGA X tool. The ITS2 haplotypes were determined manually by aligning the DNA sequences using BioEdit software (http://www.mbio.ncsu.edu/BioEdit/bioedit.html). Single nucleotide polymorphisms (SNPs) were detected and each ITS2 haplotype had its unique SNPs. Similarly, another NJ tree was constructed to assess the genetic variability among the Saudi haplotypes and 12 ITS2 *E*. *orientalis* sequences retrieved from the NCBI database. The evolutionary distances were computed using the Tamura-Nei method [23]. To support NJ tree topology, bootstrap analysis [24] with 1000 replications was conducted. The ambiguous positions for each sequence pair were removed through the pairwise deletion method.

## Results

Most variations observed in *E*. *orientalis*, collected from seven different host plants in 10 locations representing 5 regions of SA, were in the lengths and shapes of dorsal body setae especially dorso-central setae, striation pattern between setae *d1* and *e1*, and the length of legs and the chaetotaxy of leg segments; femora and tibiae (Tables 2 and 3).

**Table 2:** Phenotypic characters used in heirachical cluster analysis of *E*. *orientalis* populations collected from different hosts and regions in Saudi Arabia.

No.	Characters	States	
1	Ratio of the length to the width of idiosoma (v2-h1/c3-c3)	length almost equal to the idiosomal width	0
		length slightly longer than idiosomal width	1
		length distinctly longer than idiosomal width	2
2	Length of setae v2 vs. sc1	v2 equal in length to sc1	0
		v2 longer than sc1	1
		v2 shorter than sc1	2
3	Ratio of length and distance of setae v2 (v2/v2-v2)	half	0
		two third	1
		three quarter	2
4	length of setae sc1 vs. sc2	sc1 equal in length to sc2	0
		sc1 longer than sc2	1
		sc1 shorter than sc2	2
5	Ratio of length and distance of setae sc1(sc1/sc1-sc1)	one third	0
		half	1
6	Length of setae c1 vs. d1	c1 almost equal in length to d1	0
		c1 slightly longer than d1	1
		c1 distinctly longer than d1	2
7	Length of setae d1 vs. e1	d1 almost equal in length to e1	0
		d1 slightly longer than e1	1
		d1 distinctly longer than e1	2
		d1 distinctly shorter than e1	3
8	Length of setae e1 vs. f1	e1 almost equal in length to f1	0
		e1 slightly smaller than f1	1
		e1 distinctly smaller than f1	2
9	Length of setae f1 vs. h1	f1 almost equal in length to h1	0
		f1 slightly smaller than h1	1
		f1 distinctly smaller than h1	2
		f1 distinctly longer than h1	3
10	Ratio of setae c1/c1-c1	one third	0
		half	1
		two third	2
11	length of setae c1 vs. c2	c1 almost equal in length to c2	0
		c1 shorter in length to c2	1
12	Length of setae c2 vs. c3	c2 almost equal in length to c3	0
		c2 slightly longer than c3	1
		c2 distinctly longer than c3	2
		c2 distinctly smaller than c3	3
13	Ratio of setae c2 /c1-c2	half	0
		two third	1
		three quarter	2
14	Length of setae c3 vs. d2	c3 almost equal as d2	0
		c3 slightly longer than d2	1
		c3 distinctly longer than d2	2
		c3 distinctly smaller than d2	3
15	Length of setae c2 vs. d2	c2 almost equal in length to d2	0
		c2 slightly longer than d2	1
		c2 distinctly longer than d2	2
16	Length of setae d2 vs. e2	d2 almost equal in length to e2	0
		d2 slightly longer than e2	1
		d2 distinctly longer than e2	2
		d2 distinctly smaller than e2	3
17	Length of setae e2 vs. f2	e2 almost equal as f2	0
		e2 slightly longer than f2	1
		e2 distinctly longer than f2	2
		e2 distinctly smaller than f2	3
18	Length of setae f2 vs. h1	f2 almost as h1	0
		f2 slightly longer than h1	1
		f2 distinctly longer than h1	2
19	Ratio of setae c3/c2-c3	two third	0
		three quarter	1
		half	2
20	Ratio of setae d1/d1-d1	one sixth	0
		one third	1
21	length of setae d1 vs. d2	d1 almost equal as d2	0
		d1 slightly shorter than d2	1
		d1 distinctly shorter than d2	2
22	Ratio of setae d2/d1-d2	half	0
		two third	1
		three quarter	2
23	Ratio of e1/e1-e1	one fourth	0
		two third	1
		half	2
24	Ratio of e2/e1-e2	half	0
		two third	1
25	Ratio of f1/f1-f1	one third	0
		two third	1
		half	2
		three quarter	3
26	Ratio of setae f2/f1-f2	equal	0
		half	1
		three quarter	2
27	Ratio of setae h1/h1-h1	three quarter	0
		equal	1
28	Length of leg1 to idiosomal length (v2-h1)	leg1 almost equal to idiosomal length	0
		leg1distincly shorter than idiosomal length	1
		leg1 distinctly longer than idiosomal length	2
29	Length of leg4 to idiosomal length (v2-h1)	leg4 almost equal to the idiosomal length	0
		leg4 distinctly shorter than idiosomal length	1
		leg4 distinctly longer than idiosomal length	2
30	Length of leg2 to the idiosomal width (c3-c3)	leg2 almost equal to idiosomal width	0
		leg2 distinctly shorter than idiosomal width	1
		leg2 distinctly longer than idiosomal width	2
31	Length of leg3 to the idiosomal width (c3-c3)	leg3 almost equal to idiosomal width	0
		leg3 distinctly shorter than idiosomal width	1
		leg3 distinctly longer than idiosomal width	2
32	Length of leg1 to the length of leg4	leg1 almost equal to leg4	0
		leg1 slightly longer than leg4	1
		leg1 distinctly longer than leg4	2
33	Length of leg2 to the length of leg3	leg2 almost equal to leg3	0
		leg2 distinclty shorter than leg3	1
		leg2 distinctly longer than leg3	2
34	Stylophore	Rounded	0
		Notched	1
35	Dorsal setal shape	spatulate-subspatulate	0
		Slender	1
		slight lanceolate	2
36	Striation between the setae d1 and e1	Longitudinal	0
		V-shaped	1
37	setae on leg femora II	6 setae	0
		7 setae	1
38	setae on leg Femora III	3 setae	0
		4 setae	1
39	setae on leg Tibia I	8	0
		9	1
40	setae on leg Tibia II	5	0
		6	1

**Table 3:** Matrix data of morphological character states for Saudi *E*. *orientalis* populations.

Phenotypes										1	1	1	1	1	1	1	1	1	1	2	2	2	2	2	2	2	2	2	2	3	3	3	3	3	3	3	3	3	3	4
	1	2	3	4	5	6	7	8	9	0	1	2	3	4	5	6	7	8	9	0	1	2	3	4	5	6	7	8	9	0	1	2	3	4	5	6	7	8	9	0
**1**	1	2	2	1	1	2	2	2	1	2	1	2	2	3	0	2	0	0	0	0	3	2	1	0	3	2	1	1	1	1	1	1	0	0	1	0	1	0	0	0
**2**	2	1	2	0	0	2	0	0	2	2	0	1	1	2	2	3	0	2	2	2	0	0	2	1	2	2	1	1	1	0	1	2	2	0	0	0	0	0	0	1
**3**	1	0	2	2	0	0	0	2	2	1	1	0	1	3	2	0	1	0	0	1	2	2	2	1	1	0	1	0	0	1	1	0	0	1	1	1	1	1	1	1
**4**	1	0	2	0	0	0	2	2	2	0	1	0	0	2	1	3	0	1	0	1	1	2	0	1	1	2	1	2	1	1	0	2	1	0	0	0	0	0	0	1
**5**	2	1	2	2	0	0	0	0	2	1	1	0	0	2	0	2	3	2	0	2	3	2	2	1	0	2	1	2	2	2	2	0	2	1	0	1	-	1	1	1
**6**	0	0	1	0	0	0	0	0	2	0	1	2	2	2	2	2	0	2	1	1	3	2	3	2	2	2	1	2	2	2	2	1	0	0	0	1	0	0	1	0
**7**	0	0	2	1	1	0	1	1	3	2	1	2	2	3	2	2	2	2	0	1	2	2	3	2	3	0	0	1	1	1	1	2	1	1	2	1	0	1	1	1
**8**	2	2	1	0	0	0	0	2	3	2	1	1	0	1	1	0	3	2	1	0	2	1	3	0	3	2	1	1	1	0	1	1	0	1	0	0	1	1	1	1
**9**	1	1	0	2	0	0	3	1	2	0	1	0	1	2	0	3	2	2	1	2	1	2	2	2	1	0	1	1	1	2	1	2	0	1	0	0	0	0	1	1
**10**	0	1	2	2	0	1	0	2	2	0	1	3	0	2	2	0	0	0	0	2	1	2	0	0	0	1	0	2	2	2	1	1	0	0	0	1	0	1	1	1

**Fig 2:**
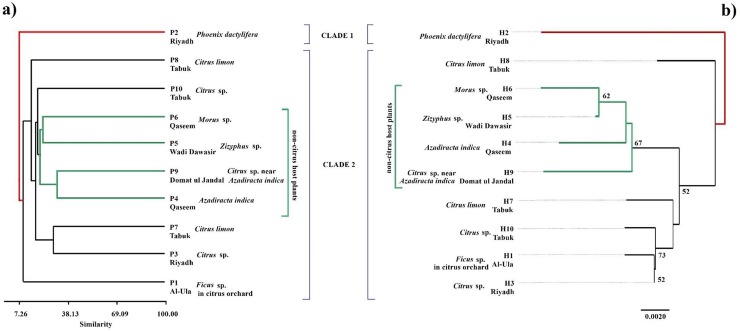
**a)** UPGMA phylogenetic tree based on different morphological characters of 10 *E*. *orientalis* phenotypes (P1-10) recovered from different hosts and regions **b)** NJ phylogenetic tree based on ITS2 sequences of *E*. *orientalis* from Saudi Arabia. Bootstrap values on the branches of the tree were generated with 1000 replicates.

In the present study, a UPGMA dendrogram generated from 40 different morphological characteristics (Fig 2A) and an NJ tree constructed based on 10 nucleotide sequences (Fig 2B) divided 10 *E*. *orientalis* populations into two main clades and nine sub clades based on host plants (monocot or dicot) rather than their geographic distribution. In both the UPGMA and NJ phylogenetic trees clade 1 contained one *E*. *orientalis* phenotype/haplotype, found on *Phoenix dactylifera* (*Arecaceae*) (a monocot) from Riyadh, with lowest phenotypic similarity percentage (6.26) and genetically highly distant from other nine haplotypes (Fig 2A and 2B). This phenotype was morphologically distinct from others by having dorso-central setae were comparatively longer and slender in shape (Table 3).

The clade 2 comprised nine phenotypes/haplotypes of *E*. *orientalis* collected from only dicot plants. Among them, four were collected from non-citrus host plants, which were clustered together in both UPGMA (sharing 10 phenotypic characters) and NJ (at 67 bootstrap value) phylogenetic trees (Fig 2A and 2B, Table 3). The phenotypes P4 (from *Azadirachta indica* (*Meliaceae*) in Qaseem) and P9 (from *Citrus* sp. (*Rutaceae*) grown within *A*. *indica* and *Morus* sp. (*Moraceae*) trees in Domat ul Jandal) showed highest affinity forming a cluster at 31.67% similarity level (Fig 2A). These two phenotypes shared half the morphological characteristics, which were used to study the phenotypic variations in the present study (Table 2). Similarly, phenotypes P5 and P6 found on *Ziziphus* sp. (*Rhamnaceae*) and *Morus* sp. from Wadi e Dawasir and Qaseem regions, respectively, joined a cluster at 22.42% phenotypic similarity level (Fig 2A) while sharing 16 phenotypic characteristics (Table 3).

Among the other five *E*. *orientalis* phenotypes/haplotypes collected from citrus host, the phenotypes P1, P8, and P10 joined in a separate sub-clade (sharing only six phenotypic characteristics), whereas P7 and P3 clustered together (sharing 13 phenotypic characteristics) showing phenotypic similarity percentage of 29.05% in the UPGMA dendrogram (Fig 2A, Table 3). However, genetically these were clustered together at 73% bootstrap value except haplotype H8, which was distantly related to all other dicot haplotypes in clade 2 (Fig 2B).

The ITS2 sequence analysis revealed that the 10 *E*. *orientalis* phenotypes gave 10 different ITS2 haplotypes. The length of ITS2 sequence ranged from 496 to 540 bps (Table 1) with an average of 38.8% G-C content. In addition, nucleotide homology among 10 haplotypes was 90–99%. The intraspecific divergence among these haplotypes ranged from 0.0020 to 0.025 (Table 4). The haplotype H3 recovered from *C*. *reticulata* in Riyadh was the least divergent (0.0020) from both H1 recovered from *Ficus* sp. planted in citrus orchard from Al-Ula (Fig 2B, Table 4) and H10 recovered from *C*. *reticulata* from Tabuk.

**Table 4:** Genetic divergence (p-distance %) of world *E*. *orientalis* ITS2 sequences reported in present study and from NCBI database.

	1. Citrus IRAN	2. Citrus IRAN	3. SPAIN	4. ISRAEL	5. ISRAEL	6. ISRAEL	7. ISRAEL	8. ISRAEL	9. ISRAEL	10. ISRAEL	11. ISRAEL	12. ISRAEL	(G1) *Ficus* sp. Al-Ula	(G2) *P*. *dactylifera* KSU, Riyadh (G2)	(G3) *Citrus* sp. Agri Farm, Riyadh	(G4) *A*. *indica* Unayzah, Qaseem	(G5) *Ziziphus* sp. Wadi Dawasir	(G6) *Morus* sp.Tabuk	(G7) *C*. *limon* Tabuk	(G8) *C*. *limon* Tabuk	(G9) *C*. *limon* Domat ul Jandal	(G10) *C*. *limon* Tabuk	DQ656454.1_ISRAEL_(palmatus_1)
1. Citrus IRAN	0.0000																						
2. Citrus IRAN	0.0039	0.0000																					
3. SPAIN	0.0045	0.0045	0.0000																				
4.ISRAEL	0.0039	0.0078	0.0045	0.0000																			
5. ISRAEL	0.0177	0.0177	0.0136	0.0138	0.0000																		
6. ISRAEL	0.0078	0.0117	0.0068	0.0078	0.0138	0.0000																	
7. ISRAEL	0.0118	0.0118	0.0068	0.0079	0.0138	0.0157	0.0000																
8. ISRAEL	0.0000	0.0039	0.0045	0.0039	0.0177	0.0078	0.0118	0.0000															
9. ISRAEL	0.0039	0.0039	0.0045	0.0039	0.0177	0.0078	0.0118	0.0039	0.0000														
10. SRAEL	0.0078	0.0039	0.0045	0.0078	0.0177	0.0117	0.0118	0.0078	0.0039	0.0000													
11. ISRAEL	0.0079	0.0079	0.0023	0.0039	0.0098	0.0118	0.0039	0.0079	0.0079	0.0079	0.0000												
12. ISRAEL	0.0059	0.0059	0.0045	0.0059	0.0118	0.0138	0.0059	0.0059	0.0098	0.0098	0.0020	0.0000											
(G1). *Ficus* sp. Al-Ula	0.0063	0.0063	0.0048	0.0063	0.0125	0.0146	0.0042	0.0063	0.0104	0.0104	0.0021	0.0000	0.0000										
(G2) *P*.*dactylifera* KSU,Riyadh	0.0274	0.0274	0.0049	0.0232	0.0254	0.0274	0.0212	0.0274	0.0274	0.0274	0.0191	0.0212	0.0212	0.0000									
(G3) *Citrus* sp. Agri Farm, Riyadh	0.0079	0.0079	0.0023	0.0039	0.0098	0.0118	0.0039	0.0079	0.0079	0.0079	0.0000	0.0020	0.0021	0.0191	0.0000								
(G4) *A*. *indica* Unayzah	0.0148	0.0148	0.0049	0.0148	0.0170	0.0148	0.0127	0.0148	0.0148	0.0148	0.0106	0.0127	0.0127	0.0193	0.0106	0.0000							
(G5) *Ziziphus* sp. Wadi Dawasir	0.0062	0.0062	0.0024	0.0062	0.0125	0.0104	0.0104	0.0062	0.0062	0.0062	0.0062	0.0083	0.0083	0.0211	0.0062	0.0085	0.0000						
(G6) *Morus* sp.Tabuk	0.0061	0.0102	0.0071	0.0061	0.0163	0.0102	0.0143	0.0061	0.0061	0.0102	0.0102	0.0122	0.0125	0.0253	0.0102	0.0085	0.0041	0.0000					
(G7) *C*. *limon* Tabuk	0.0131	0.0131	0.0025	0.0087	0.0153	0.0131	0.0066	0.0131	0.0131	0.0131	0.0044	0.0066	0.0066	0.0174	0.0044	0.0109	0.0109	0.0153	0.0000				
(G8) *C*. *limon* Tabuk	0.0146	0.0146	0.0024	0.0105	0.0168	0.0188	0.0084	0.0146	0.0146	0.0146	0.0063	0.0084	0.0084	0.0169	0.0063	0.0170	0.0126	0.0167	0.0109	0.0000			
(G9) *C*. *limon* Domat ul Jandal	0.0149	0.0149	0.0099	0.0149	0.0171	0.0170	0.0128	0.0149	0.0149	0.0149	0.0107	0.0128	0.0128	0.0234	0.0107	0.0108	0.0085	0.0128	0.0131	0.0171	0.0000		
(G10) *C*. *limon* Tabuk	0.0098	0.0098	0.0023	0.0059	0.0118	0.0138	0.0059	0.0098	0.0098	0.0098	0.0020	0.0039	0.0042	0.0212	0.0020	0.0127	0.0083	0.0122	0.0066	0.0084	0.0128	0.0000	
*E*. *palmatus* ISRAEL	0.0568	0.0527	0.0584	0.0545	0.0467	0.0527	0.0508	0.0568	0.0525	0.0525	0.0508	0.0528	0.0518	0.0658	0.0508	0.0549	0.0516	0.0549	0.0567	0.0563	0.0509	0.0528	0.0000

The yellow highlighted sections indicate three haplotypes with zero p-distance percentage

*The blue highlighted section represents the divergence values of E*. *palmatus (DQ656451*.*1 from Israel) used as an outgroup in present study*.

**Accession numbers of E. orientalis genotypes collected from GenBank database and used in present study are as follows:**

**1.** HQ688670, **2.** HQ585023, **3.** KP642048, **4.** DQ656479, **5.** DQ656478, **6.** DQ656477, **7.** DQ656476, **8.** DQ656473, **9.** DQ656472, **10.** DQ656470, **11.** DQ656474, **12.** DQ656471

**Fig 3:**
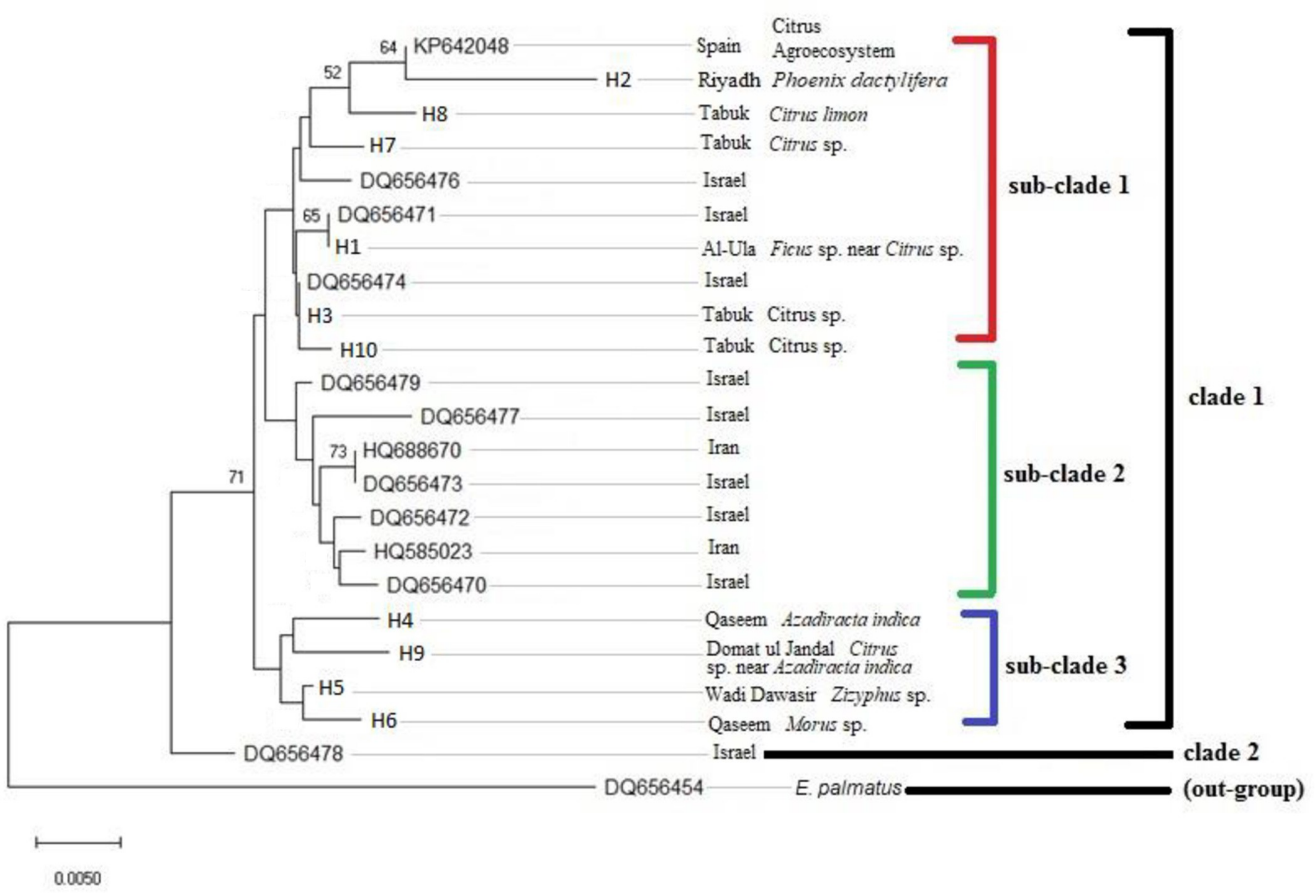
NJ tree based on ITS2 sequences obtained from 10 *E*. *orientalis* haplotypes recovered from different hosts and regions in Saudi Arabia, and 12 homologous ITS2 sequences retrieved from NCBI-GenBank database. The ITS2 sequence DQ656454 *E*. *palmatus*, a genetically close species to *E*. *orientalis*, was used as an out-group taxon. Numbers on the tree branches are bootstrap values obtained from 1000 replicates.

The NJ phylogenetic tree based on 22 ITS2 sequences of *E*. *orientalis* haplotypes from different countries including those recovered in the present study showed that most haplotypes were generally clustered based on host plant association rather than geographic distribution. (Fig 3). Haplotypes H1 and H3 were 100% identical to *E*. *orientalis* ITS2 haplotypes DQ656471 and DQ656474, previously collected from Israel on *Citrus* sp., respectively (Table 4, Fig 3). Further, two *E*. *orientalis* haplotypes, HQ688670 from Iran (host plant unknown) and DQ656473 from Israel, were 100% identical to each other (Table 4, Fig 3). The haplotype H2 from *P*. *dactylifera* (*Arecaceae*), which was genetically divergent (0.016–0.025) from all other *E*. *orientalis* haplotypes, clustered with a Spanish haplotype (KP642048) from citrus agro-ecosystem in the NJ phylogenetic tree (Table 4, Fig 3).

## Discussion

In the present study, the phenotypic variations among 10 Saudi *E*. *orientalis* populations collected from different hosts and regions of SA were found mostly in lengths and shapes of dorsal body setae especially dorso-central setae, striation pattern between setae *d1* and *e1*, and length of legs and chaetotaxy of leg segments; femora and tibiae (Tables 2 and 3). These variations have been well documented in different *E*. *orientalis* populations from different countries [5, 10] and have resulted in the addition of species in the genus *Eutetranychus* [4]. Because of these intraspecific phenotypic variations in *E*. *orientalis* populations, four *Eutetranychus* species viz. *E*. *ricini* [25], *E*. *monodi* [26], *E*. *sudanicus* [27], and *E*. *annecki* [1,7,8] were synonymized with *E*. *orientalis*. Recently, a study was conducted [4] on the phenotypic variations among different populations of *E*. *orientalis* from SA and it reported seven species, viz. *E*. *phaseoli* [28], *E*. *guangdongensis* [29], *E*. *xianensis* [29], *E*. *fici* [8], *E*. *pruni* [30], *E*. *ricinus* [30], and *E*. *sanaae* [30] as junior synonyms of *E*. *orientalis*. The present study fortifies the findings of a previous study [4] and the comprehensive diagnostic key provided should be followed for the proper identification of *Eutetranychus* species.

The present study revealed that different hosts associated with *E*. *orientalis* races could be present, which might cause phenotypic and genetic differences in these races. Further, *E*. *orientalis* is a polyphagous species reported from > 223 diversified host plants including shrubs and trees from both mono and dicot plant families [1]. Host-associated phenotypes have frequently been found in other phytophagous arthropods mainly pest insects [31–33] and spider mites i.e. *Tetranychus urticae* Koch [34–36, 13] and *Panonychus citri* (McGregor) [37]. Populations from closely related hosts show closer relationships. It has been reported that the species of spider mites, like *T*. *urticae*, *T*. *kanzawai*, *and Oligonychus gotohi* adapted to different hosts were reproductively isolated and genetically different [35,38,39]. Similar results were reported in a previous study [36], that *T*. *urticae* populations from the same host *Viola* sp. from Italy and France were more closely related when compared with those from different hosts from the same country. The species, *Brevipalpus phoenicis*, which is a host specific species, failed to adapt on alternate hosts and did not exhibit host based genetic variations [40]. However, this species had significant genetic variability among/between populations when collected and compared from different localities in Mexico and Brazil [41].

The utilization of different host plants may cause sympatric speciation in arthropods [42]. The phytophagous species and plants exert strong selective pressures on each other [43]. In addition, herbivores when exposed to and colonized on a new host species, resist and/or tolerate defense mechanisms specific to that new host, which results in either adaptation or local extinction of the phytophagous species due to strong selection pressure [44]. Spider mites inhabiting wide host range like *T*. *urticae* may form host races to combat toxic chemicals produced by different hosts influencing high selection pressure on herbivores to provide opportunity for prolonged existence on different hosts and strong association [45].

In the present study, the genetic data of 10 Saudi *E*. *orientalis* populations was associated with host plants they inhabited (Fig 2A and 2B). The phylogenetic tree based on world *E*. *orientalis* ITS2 sequences, including from the present study, supported host association (Fig 3). Similar findings were reported in *Tetranychus urticae* (Koch) [36] and *Amphitetranychus viennensis* (Zacher) [46]. In a previous study, nine *E*. *orientalis* populations from Israel have been reported as genetically distinct [10]. The intraspecific genetic divergence of nine Saudi *E*. *orientalis* haplotypes was in a similar range (0.002 to 0.017) as found in the Israeli *E*. *orientalis* haplotypes [10].

The Saudi haplotype H1 collected from *Ficus* sp. in a citrus orchard was 100% identical to Israeli haplotype (DQ656474 from *Citrus* sp.) and shared the clade with other Saudi haplotypes collected from *Citrus* sp. Likewise, the genotype H9 from Domat ul Jandal on *Citrus* sp. under the *A*. *indica* trees was clustered in the phylogenetic tree and dendrogram with G4 from *A*. *indica* from Qaseem; this might be due to the colonization of *E*. *orientalis* on a new host. The herbivores adapt on new hosts with the passage of time after coping with the defense mechanisms of new hosts [44, 47]. The wide range of host plants and geographic distribution of *E*. *orientalis* indicate that this species has a strong ability to adopt to diversified hosts and cope with defenses exerted by the host species, thus making it possible to live successfully in different environmental conditions and topography. The mite species occurring on different host plants undergo marked changes in their behavior, morphology, and genetic structure. This commonly happens in polyphagous mite species where adaptation to different host plants results in genetic differentiation [48]

In addition, the intraspecific genetic variations in mites could also be due to geographic distances. The geographic distances act as a barrier for gene flow and promote intraspecific differentiations. The mite species, *Lepidoglyphus destructor*, which have active dispersal capabilities, have shown genetic differentiations among populations collected from localities that are far apart from each other [49]. In the case of grapevine bud mites, which have limited dispersal, strong genetic differentiation was reported among the specimen collected from different vineyards and even different parts of same vineyard [50]. The genetic diversity was also recorded in the Citrus mite, *Panonychus citri*, collected from different localities in China [51].

## Conclusion

The polyphagous behavior and wide distribution of *E*. *orientalis* resulted in variations in phenotypic characteristics as well as genetic structures. The taxonomists should consider the phenotypic variations specially in the shape and length of dorsal setae and striation pattern between dorsal setae *d1* and *e1*, while identifying different *E*. *orientalis* populations to avoid improper identifications. Further extensive studies are needed to investigate the occurrence of different races of *E*. *orientalis* associated with a specific host or locality. Also, its population structure needs consideration in different agro-ecosystems.

## Supporting information

S1 Fig 1.2% agarose gel stained with 1 μg/ml acridine orange dye in 1× TAE buffer showing PCR products of ITS2-rDNA region generated from 10 *eutetranychus orientalis* samples representing 10 different mite populations collected from Saudi Arabia. (DOCX)

S1 Table DNA quantification, using nanodrop, of 10 *eutetranychus orientalis* samples representing different mite populations collected from five regions in Saudi Arabia. (DOCX)
