# Supplementary material for: Molecular and phenotypic variations in Eutetranychus orientalis (Klein) populations from Saudi Arabia
Source: PLoS One. 2020 May 19;15(5):e0233389. doi: 10.1371/journal.pone.0233389 (PMC7237003; doi:10.1371/journal.pone.0233389)
Supplement: S1 Fig — (DOCX) [file pone.0233389.s001.docx]

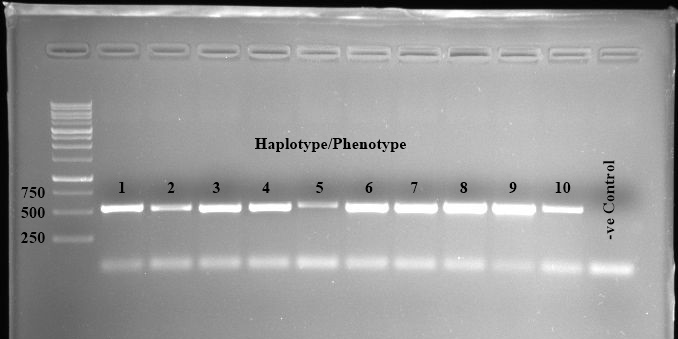


**Figure 1S.** 1.2% agarose gel stained with 1 μg/ml acridine orange dye in 1× TAE buffer showing PCR products of ITS2-rDNA region generated from 10 *Eutetranychus orientalis* samples representing 10 different mite populations collected from Saudi Arabia
