# Supplementary material for: Molecular and phenotypic variations in Eutetranychus orientalis (Klein) populations from Saudi Arabia
Source: PLoS One. 2020 May 19;15(5):e0233389. doi: 10.1371/journal.pone.0233389 (PMC7237003; doi:10.1371/journal.pone.0233389)
Supplement: S1 Table — (DOCX) [file pone.0233389.s002.docx]

**Table 1S.** DNA quantification, using Nanodrop, of 10 *Eutetranychus orientalis* samples representing different mite populations collected from five regions in Saudi Arabia

| **Haplotype (H) Phenotype (P)** | **Locality/Region** | **Nucleic Acid Conc.** ng/µl | **A_260_** | **A_280_** | **A_260_/A_280_** | **A_260_/A_230_** |
| --- | --- | --- | --- | --- | --- | --- |
| 1 | Al-Ula/ Medinah | 19.6 | 0.392 | 0.44 | 0.89 | 0.13 |
| 2 | King Saud University/ Riyadh | 14.1 | 0.282 | 0.288 | 0.98 | 0.14 |
| 3 | Agricultural Farm/ Riyadh | 13 | 0.261 | 0.255 | 1.02 | 0.15 |
| 4 | Unayzah/ Qaseem | 14 | 0.279 | 0.281 | 0.99 | 0.15 |
| 5 | Wadi Dawasir/ Riyadh | 15.3 | 0.307 | 0.304 | 1.01 | 0.15 |
| 6 | Unayzah/ Qaseem | 13 | 0.261 | 0.272 | 0.96 | 0.14 |
| 7 | TADCO/ Tabuk | 15.7 | 0.313 | 0.309 | 1.01 | 0.17 |
| 8 | Al-Bir/ Tabuk | 15.6 | 0.312 | 0.285 | 1.09 | 0.2 |
| 9 | Domat ul Jandal/ Al-Jawf | 13 | 0.261 | 0.253 | 1.03 | 0.17 |
| 10 | TADCO/ Tabuk | 15.6 | 0.313 | 0.283 | 1.1 | 0.18 |
